# Our experience regarding parametric assistance and modulation of physical interventions for the recovery of effort capacity in patients after acute myocardial infarction – partial results

**DOI:** 10.25122/jml-2026-0040

**Published:** 2026-05

**Authors:** Mihaela Mandu, Gelu Onose, Catalina Liliana Andrei, Andreea Elena Lacraru, Ioana Andone, Alexandru Ion, Dragos Alin Trache, Liliana Florina Andronache, Ana Maria Vasile, Stefan Sebastian Busnatu

**Affiliations:** 1 Carol Davila University of Medicine and Pharmacy, Bucharest, Romania; 2 Bagdasar-Arseni Clinical Emergency Hospital, Bucharest, Romania

**Keywords:** cardiovascular rehabilitation (CVR), moderate-intensity continuous training (MICT), sensor ergometric bicycle, acute myocardial infarction (acute MI), Acute MI, acute myocardial infarction, BMI, body mass index, CVR, cardiovascular rehabilitation, SBP, systolic blood pressure, DBP, diastolic blood pressure, max HR, maximum heart rate, ECG, electrocardiogram, LVEF, left ventricular ejection fraction, LOAD, resistive load, METs, metabolic equivalents, MICT, moderateintensity continuous training, M, male gender, F, female gender, Age Cat, age category, 95% CI, 95% confidence interval, *P*, *p*-value, σ^2^, residual variance (intraindividual variance), τ00 ID, random intercept variance, ICC, intraclass correlation coefficient, N (ID), number of patients, GVIF, generalized variance inflation factor, VIF, variance inflation factor, VO_2_max, maximal oxygen consumption

## Abstract

The prescription of physical exercise and the individualization of cardiovascular rehabilitation (CVR) programs after acute myocardial infarction (MI) are based on identifying the degree of patients’ deconditioning to physical effort. This bicentric, prospective, longitudinal, interventional, non-randomized, pilot study included 44 patients with acute MI who underwent at least eight sessions of moderate-intensity continuous training (MICT) on a sensorized ergometer bicycle. The main aim of the study was to determine whether energy consumption and metabolic equivalents (METs) improved during MICT sessions within the RCV program. The average energy consumption increased progressively from 223.8 ± 125.97 kJ to 407.91 ± 189.87 kJ (*P* < 0.001), with significant differences starting with moment III to moment VIII, an evolution also reflected by the METs values, which increased from 3.47 ± 1.12 to 4.71 ± 1.39, becoming significant starting with moment III, the statistical significance being consolidated starting with moment IV in the final model. Male gender and maximum heart rate (max HR) were positively associated with both energy consumption and MET values. Left ventricular ejection fraction (LVEF) was significantly associated only with energy consumption (β = 4.52; P = 0.003), while for METs it showed only a tendency toward significance (β = 0.03; *P* = 0.064). Systolic blood pressure (SBP) was significantly associated only with METs (β = 0.01; *P* < 0.001). Interindividual variability was significant for both energy consumption and METs, with intraclass correlation coefficients (ICC) of 0.77 and 0.74, respectively. The increase in energy efficiency observed within the program suggests that patients presented a favorable cardiovascular adaptation to exercise and, implicitly, an improvement in both the level and efficiency of physical effort.

## INTRODUCTION

According to the most recent recommendations of international scientific associations, including the American College of Cardiology (ACC) and the American Heart Association (AHA), cardiovascular rehabilitation (CVR) is an essential component in the management of patients with acute coronary syndromes. It is integrated into secondary prevention strategies and, from the perspective of medical rehabilitation, represents a portfolio of interventions aimed at preventing or minimizing the development of chronic, irreversible dysfunctions or disabilities; therefore, it also contributes to tertiary prevention in cardiovascular diseases. The indication to refer patients after myocardial infarction (MI) to a specialized cardiovascular rehabilitation program is classified as a Class I recommendation, with the highest level of evidence (Level A), taking into account the demonstrated benefits in reducing mortality, decreasing the frequency of relapses or rehospitalizations, and increasing functional performance, as well as improving life quality [1].

The current model of cardiovascular rehabilitation (CVR) is based on a multimodal, interdisciplinary, and multiprofessional approach that integrates several essential, interrelated components (physical, educational, and psychosocial). According to international guidelines, the basic elements of modern CVR programs include the development and the prescription of the supervised physical training rehabilitation program, the initial complex assessment of the patient, the counseling or the education regarding retraining to physical effort and adherence to treatment and rehabilitation on a medium and long term, related nutritional interventions and lifestyle correction, the management of cardiovascular risk factors (control of body weight, blood pressure, heart rate, the correction of lipid profile, the treatment of metabolic syndrome/ diabetes mellitus, smoking cessation), the proactive motor behavior, support and possibly psychosocial interventions [2].

Despite the well-documented benefits of CVR [3], the specialized literature includes relatively few studies that examine the dynamics of functional parameters within CVR programs, most of which evaluate results before and after completion of the programs rather than the evolution of energy consumption across consecutive aerobic training sessions.

The main objective of the study was to determine whether energy consumption and metabolic equivalent (MET) values improved during moderate-intensity continuous training (MICT) sessions within the CVR program.

The secondary objective of the study was to identify some factors associated with this energy consumption.

The study hypothesis was that energy consumption and MET values would increase during the MICT sessions within the CVR program compared with the initial assessment. This increase was expected to have a marked individual component, with differences in the progression pattern observed among patients.

## MATERIAL AND METHODS

### The description of the CVR program

**Figure 1:**
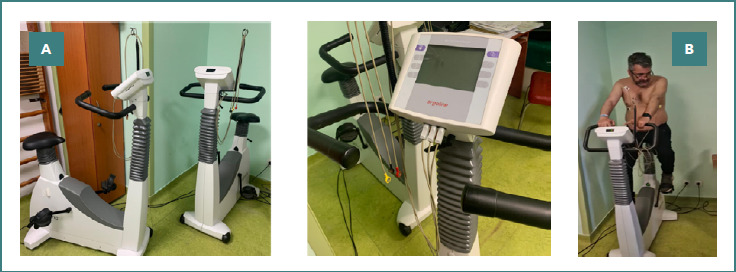
Sensor ergometric bicycle used during the cardiovascular rehabilitation program. A, Sensor ergometric bicycles used for training and monitoring during the CVR program. B, First patient undergoing MICT in the Rehabilitation Clinic of “Bagdasar-Arseni” Clinical Emergency Hospital, Bucharest, Romania.

The CVR program was developed and carried out through the collaboration between the Clinical Department of Cardiology and the Clinical Department of Neuromuscular Rehabilitation of “Bagdasar-Arseni” Clinical Emergency Hospital (BACEH). The CVR program consisted of MICT training sessions, carried out under medical supervision (through outpatient/ongoing hospitalization), using ergometric bicycles equipped with computerized systems that allow the evaluation and adaptation of functional parameters in real time, thus facilitating objective and standardized monitoring of physical effort (Figure 1AB). To ensure data reproducibility, all assessments were performed according to a standardized protocol using the same ergometric bicycles (Ergoline model, manufacturer BTL).

It has special software that allows the objective quantification of the body’s response to physical effort by measuring certain parameters recorded for every session, and the testing conditions (for example, time of day, type of effort) were kept as constant as possible.

The patients in the study underwent at least eight MICT sessions.

### The description of the training sessions

Each training session included a warm-up phase, an effort phase, and a recovery phase, with an average total duration of roughly 20–25 minutes, adapted to each individual.

The initial intensity of physical effort was set at approximately 60% of the patient’s maximum heart rate during the simple effort test. All patients included in the study were clinically and paraclinically evaluated by the cardiologists at “Bagdasar-Arseni” Clinical Emergency Hospital in Bucharest using a simple effort test.

**Figure 2:**
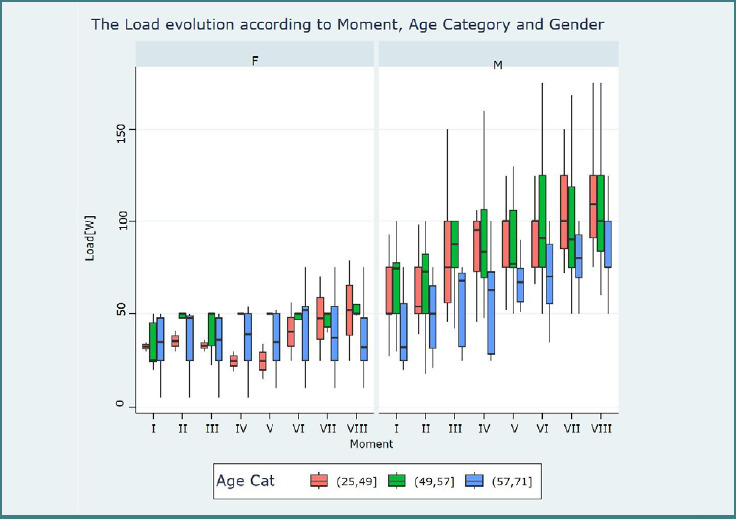
The distribution of resistive load (LOAD) values according to the assessment moment, age category, and gender

The workload was adjusted progressively according to each patient’s tolerance. Protocol adaptation considered symptoms, compliance with exercise, fatigue, psycho-emotional status, electrocardiographic (ECG) changes, and clinical parameters monitored during exercise, including maximum heart rate (max HR) and blood pressure (BP). Thus, exercise intensity was periodically modulated in accordance with the patient’s individual response and clinical evolution (Figure 2).

All the patients followed the physical training recommendations on the ergometric bicycle presented in Table 1.

**Table 1:** Physical training recommendations of the ergometric bicycle

Stages	Intensity	Duration	Frequency
**The initial stage**	Low: 40%-50% of maximal oxygen consumption (VO_2_max); 60% of max heart rate (max HR) 40% cardiac frequency upon rest; below the first ventilatory threshold; Modified Borg scale score < 4	Starts at 5 minutes during the active exercise phase and gradually increases to 10 minutes	3-5 days/week; preferable daily
**The progression stage**	Gradual and individualized increase, towards target levels: 60-80% max VO_2_; 80-85% max HR; 65-70% cardiac frequency upon rest; Modified Borg scale score: 5-7	Gradual prolongation of the training session to 20-40 minutes	3-5 days/ week; preferable daily
**The maintenance stage**	Long-term maintenance of intensity and duration of effort; gradual increase, if well tolerated	Gradual prolongation of the duration, from 40 minutes to more than 60 minutes, if well tolerated; preferable increase in duration before increase in intensity	3-5 days/ week; preferably on most weekdays

The modulation of physical effort for each training session was done according to the criteria presented in Table 2, which has an indicative character. In practice, the intensity and the duration of training were individualized according to the patient’s overall clinical and functional aspects, including both objective elements such as the appearance of cold sweats, pallor, cyanosis, dyspnea, blood pressure values Thabove the allowed thresholds, electrocardiographic (ECG) signs suggestive of ischemia, and subjective aspects such as inadequate mood, lack of desire or motivation to continue training, episodic psycho-emotional disorders, which can equally significantly and decisively influence the patient’s adherence to retraining for physical exertion. In modulating therapeutic interventions, we also considered that a patient with a normal left ventricular ejection fraction (LVEF) can tolerate a certain heart rate. In contrast, in a patient with severely reduced LVEF, the same value may be associated with the appearance of manifestations of cardiac decompensation (e.g., acute pulmonary edema).

**Table 2:** Criteria for the adjustment of LOAD and effort interruption during MICT sessions

Domain	Parameter	Criteria to increase LOAD	Criteria to reduce or interrupt effort
**Symptoms**	Thoracic pain, dyspnea, fatigue	Absence or lack of worsening cardiac failure symptoms (according to New York Heart Association (NYHA) classification) [4]	The occurrence/aggravation of cardiac failure symptoms (according to the NYHA classification)
**ECG**	Ischemic cardiac rhythm and changes	No significant arrhythmias or ischemic modifications	Significant arrhythmias (e.g., sustained or non-sustained ventricular tachycardia, newly emerged atrial fibrillation, severe bradycardia) or changes indicative of ischemia (ST segment elevation)
**Hemodynamic response**	Blood pressure	Adequate increase in BP during effort	Hypotension or excessively increased blood pressure (e.g., SBP > 220 mmHg or DBP > 110 mmHg) [5]
**Cardiac frequency**	Response to effort	Preserved within the target interval (60-85% of max HR reached during the effort test) according to the stage	Inadequate response (decrease in HR)
**Effort perception**	Score on the modified Borg scale	Moderate effort (Borg score 3-4)	Increased perceived effort (score Borg >7-8)
**LOAD adjustment**	LOAD	Gradual increase	Intensity reduction or effort cessation, depending on the tolerance level expressed subjectively or objectively (hyper-/hypotension, arrhythmias, vegetative disorders indicative of ischemia – pallor, profuse sweating, etc.)

Patient monitoring during training was continuous via ECG, including periodic heart rate and blood pressure measurements.

### The study design

We developed a bicentric, prospective, longitudinal, experimental, non-randomized, pilot study (see below). The study design included repeated daily measurements for each patient, with eight energy consumption and MET measurements recorded during the MICT sessions. The time points were defined as follows: Moments I–VII correspond to the first seven consecutive training sessions for each patient, and Moment VIII represents the last training session during the rehabilitation program, regardless of the total number of sessions. Given the variability in program duration among patients, Moment VIII does not necessarily correspond to the 8^th^ session. Thus, the analysis included the first seven sessions and the last available session for each participant.

### The population sample and the inclusion criteria

Patients were enrolled prospectively through consecutive eligible recruitment during the study period. All participants followed the standard medication treatment after the acute myocardial infarction, according to valid guidelines, with adjustments, all performed by the cardiologist.

Patients who met the inclusion and exclusion criteria (Table 3) were assessed in the cardiology department. Patients with acute myocardial infarction (MI), with or without ST-segment elevation, were included in the study at least 1 week after the supra-acute episode, provided that they had a permissive exercise test.

**Table 3:** Inclusion and exclusion criteria for the patients included in the study

INCLUSION CRITERIA	EXCLUSION CRITERIA
Confirmed diagnosis of acute MI	Patients with cardiovascular pathology, other than acute MI
Patients who underwent two effort tests before and at least within 3 months after the completion of CVR sessions	Patients who did not undergo the two effort tests at the beginning and within at least 3 months after the completion of CVR sessions
Clinical and biological stability at the beginning of the CVR program	Patients who are clinically and biologically unstable at the time of testing
Informed consent was signed for participation in the study	Patients who did not sign the informed consent for participation in the study
The patients must have undergone at least 8 days of CVR through training on the sensor ergometric bicycle	The patients who underwent fewer than 9 days of CVR on the sensor ergometric bicycle

**Figure 3:**
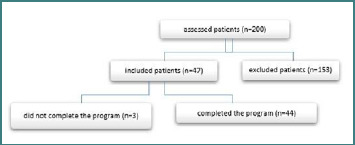
The diagram of the selection flow of patients

In total, 200 patients were assessed for eligibility, out of which 47 were initially included in the CVR program. 44 patients completed the program and were included in the final analysis (Figure 3); for 39 of them, METs were also calculated.

The analyzed group (*n* = 44) had a mean age of 54 ± 8 years and a relatively balanced distribution around the median. A total of 35 patients were men (80%) and 35 women (20%), as highlighted in Table 4.

**Table 4:** Basic characteristics of the sample

Variable	*n* = 44
**Age (years)**	
Average (SD)	54 (8)
Median (IQR)	54 (11)
Min to Max	41 to 71
**Gender, *n* (%)**	
F	9 (20)
M	35 (80)
**Smoking, *n* (%)**	
Yes	26 (59)
No	18 (41)
**LVEF (%)**	
Average (SD)	44 (10)
Median (IQR)	45 (15)
Min to Max	25 to 68
**BMI (kg/m^2^)**	
Average (SD)	28.8 (4.4)
Median (IQR)	27.7 (5.0)
Min to Max	20.4 to 39.1
**Age Cat (years), *n* (%)**	
(25,49)	14 (32)
(49,57)	18 (41)
(57,71)	12 (27)
**STEMI, *n* (%)**	
Non-STEMI	4 (11)
STEMI	31 (89)
N/A	9
**Days after acute MI**	
Average (SD)	117 (251)
Median (IQR)	44 (49)
Min to Max	11 to 1,160
N/A	6
**NYHA, *n* (%)**	
I	1 (8)
II	10 (84)
III	1 (8)
N/A	32
**Max HR (bpm)**	
Average (SD)	110 (24)
Median (IQR)	104 (15)
Min to Max	79 to 196

A significant percentage of patients were smokers (59%). In terms of cardiac functional status, the LVEF averaged 44%, indicating mild to moderate ventricular dysfunction in most cases. The average body mass index (28.8 kg/m^2^) suggested a high prevalence of overweight patients. Most patients underwent acute myocardial infarction with ST-segment elevation (STEMI – 89%). The high proportion of missing data limits the interpretation of the NYHA functional class; however, among the patients assessed, class II predominates, indicating moderate impairment of functional capacity. The maximum HR values recorded during effort indicate moderate variability, compatible with a low to moderate functional level. The time interval from the acute MI to the implementation of the rehabilitation program shows very high variability, with values Th≥11 days, according to the applied protocol.

### The definition of parameters and the means through which they were achieved

The dependent variables were energy consumption, expressed in kilojoules (kJ), and METs. The independent variables included baseline variables measured before the start of the physical exercise program, namely age category, gender, body mass index (BMI; kg/m^2^), left ventricular ejection fraction (LVEF; %), and tobacco consumption, as well as variables measured during each exercise session, including systolic blood pressure (SBP; mmHg), diastolic blood pressure (DBP; mmHg), maximum heart rate (max HR; bpm), resistive load (LOAD; W), and the number of aerobic training sessions. Maximum heart rate (max HR) was recorded during training using the integrated sensors and expressed in beats per minute (bpm). Resistive load (LOAD) was measured in watts (W) by the ergometric bicycle software during aerobic training, and its maximum value was included in the present analysis. The effort duration was expressed in minutes, and the intensity was adjusted based on real-time parameters. All measurements were performed using the same equipment under standardized conditions for all participants. LVEF [%] was measured through echocardiography, using the biplane Simpson method, before the beginning of the CVR program. Systolic blood pressure (SBP) and diastolic blood pressure (DBP) were expressed in mmHg and measured using an electronic blood pressure monitor, independent of the sensor ergometer, with maximum values recorded during physical effort for each training session. At the beginning of each MICT session, target values for LOAD and max HR were set.

The study’s outcome variables were energy consumption and METs during physical training.

The software connected to the sensor bicycles permitted the direct calculation of these two variables during the training sessions. The ergometric bicycle was not equipped with a metabolic cart that included oxygen and carbon dioxide analyzers. Through this method, the objective measurement of mechanical effort during each training session was achieved.

### Statistical analysis

The R program was used for statistical analysis, version 4.5.1, (c) 2025 (The R Foundation for Statistical Computing). The alpha level of the study was 0.05; *P* values below 0.05 were considered statistically significant.

To assess the relationship between the analyzed predictors and energy consumption, and respectively METs, a linear, simple, multilevel mixed-effect model (with random effects) was designed, the effects being measured at the population level – at the level of the study group (fixed effects) – applied to the entire sample, as well as at the group level – at the level of each individual (“random” effects) – applied to each patient. A complete multiple model was also performed, which proved to be representative for this study.

Random intercepts were used because each patient started at a different energy level. Given the sample size, the analysis by age group was conducted using broader categories.

The results of the models were reported as regression coefficients and 95% confidence intervals (CIs). *P* values <0.05 were considered statistically significant.

Robust statistical methods were used to reduce the influence of extreme values (outliers) on the results. However, residual factors cannot be completely excluded. No imputation procedures were performed for the missing data; the analysis was based on the available data.

## RESULTS

### Results regarding the energy consumption of patients

The comparison of the models highlights differences in the regression coefficients between the simple and multiple models, both in magnitude and statistical significance, as shown in Table 5.

A backward selection procedure was used to construct the multiple regression model, based on P values and collinearity indices, including the variance inflation factor (VIF) and adjusted VIF (Table 6). We attempted to construct a multiple model in which all predictors had low *P* values (e.g., below 0.10). A more permissive threshold (*P* < 0.10) was adopted in the selection stage to avoid premature exclusion of potentially relevant predictors, with final statistical significance assessed at the standard threshold of *P* < 0.05.

**Table 5:** Simple models vs. complete multiple models regarding energy consumption

Predictor	Simple models			Complete multiple model			
	*n*	Beta (95% CI)	*P* value	Beta (95% CI)	*P* value	GVIF	Adjusted GVIF1
Moment	352					1.2	1.0
I		—		—			
II		13(-8. to 35)	0.22	15 (-7 to 36)	0.18		
III		36 (15 to 58)	0.001	32 (10 to 54)	0.005		
IV		50 (28 to 72)	<0.001	47 (25 to 69)	<0.001		
V		80 (58 to 101)	<0.001	74 (52 to 96)	<0.001		
VI		98 (76 to 119)	<0.001	89 (67 to 112)	<0.001		
VII		98 (77 to 120)	<0.001	91 (69 to 113)	<0.001		
VIII		148 (127 to 170)	<0.001	140 (117 to 162)	<0.001		
Age (years)	352	-7.5 (-12 to -3.)	0.001	-1.5 (-6 to 3)	0.49	1.4	1.2
Gender	352					1.3	1.2
F		—		—			
M		177 (95 to 260)	<0.001	172 (90 to 255)	<0.001		
LVEF (%])	352	2.7 (-1.44 to 7)	0.19	4.2 (1 to 7.3)	0.011	1.1	1.1
BMI (kg/m^2^)	352	2.7 (-7 to 12)	0.57	1.64 (-5 to 8)	0.63	1.1	1.0
Max HR (bpm)	352	1.8 (1.4 to 2.2)	<0.001	0.8 (0.4 to 1.2)	<0.001	1.1	1.1
SBP (mmHg)	352	-0.4 (-1.2 to 0.4)	0.28	-0.3 (-1 to 0.4)	0.37	1.4	1.2
DBP (mmHg)	352	-0.05 (-1 to 0.9)	0.91	0.3 (-0.6 to 1.2)	0.49	1.4	1.2

1 GVIF^[1/(2*df)] Abbreviations: CI, Confidence Interval; GVIF, Generalized Variance Inflation Factor.

The model accounts for approximately 88% of the outcome variance, with approximately 47% explained by fixed effects (Marginal R^2^ = 0.466) and approximately 41% by random effects (Conditional R^2^ = 0.875). Interindividual variability was significant (ICC = 0.77).

### The influence of the moment on the energy consumed during MICT

According to the final multiple model (Table 6), energy consumption increased progressively throughout the training sessions. Compared to the initial moment, the difference was not statistically significant for the 2^nd^ session (β = 14.36; CI 95%, -7.23 to 35.95; *P* = 0.192), but it became statistically significant starting with the 3^rd^ session(β = 31.66; CI 95%, 9.86 to 53.46; *P* = 0.005), maintaining and progressively increasing up to the 8^th^ session (β = 139.64; CI 95%, 117.44 to 161.84; *P* < 0.001).

**Table 6:** The final multiple model for energy consumption, including significant predictors

	Energie [kJ]		
Predictors	Beta (kJ)	CI 95%	*P*
Moment [II]	14.36	-7.23 to 35.95	0.192
Moment [III]	31.66	9.86 to 53.46	0.005
Moment [IV]	47.32	25.60 to 69.05	<0.001
Moment [V]	73.86	51.99 to 95.72	<0.001
Moment [VI]	88.47	66.35 to 110.60	<0.001
Moment [VII]	91.57	69.60 to 113.55	<0.001
Moment [VIII]	139.64	117.44 to 161.84	<0.001
Gender [M]	185.10	113.84 to 256.36	<0.001
LVEF [%]	4.52	1.55 to 7.48	0.003
Mac HR [bpm]	0.80	0.42 to 1.18	<0.001
**Random Effects**			
σ^2^	2519.19		
τ_00__ID_	8263.78		
ICC	0.77		
N _ID_	44		
Observations	352		
Marginal R^2^ / Conditional ^R^2	0.466 / 0.875		

The same progressive increase was also obvious when using descriptive data (Table 7), where the mean increased from 223.8 ± 125.97 kJ at the starting point (I) to 407.91 ± 189.87 kJ at the final time (VIII), representing a mean energy increase of approximately 184.10 kJ. A similar trend was observed at the mean level, which increased from 216.5 kJ at moment I to 376.5 kJ at moment VIII, suggesting a consistent improvement in energy consumption among the entire group.

**Table 7:** The evolution of energy consumption – descriptive data

	Moment	*n*	Average	SD	Median	Minimum	Maximum
1	I	44	223.8	125.97	216.5	2	570
2	II	44	239.27	125.12	202.5	7	580
3	III	44	263.02	116.65	244.5	5	533
4	IV	44	280.2	125.51	265.5	3	530
5	V	44	309.07	136.62	298.5	18	613
6	VI	44	328.95	145.2	318	14	650
7	VII	44	331.34	148.2	325	18	646
8	VIII	44	407.91	189.87	376.5	17	898

The minimum and maximum values indicate significant variability among patients throughout the program.

**Figure 4:**
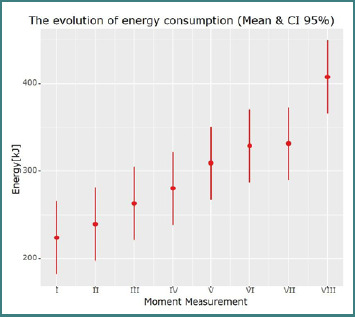
The evolution of energy consumption throughout the CVR program (mean and confidence interval of 95%)

In the graph shown in Figure 4, energy consumption increased progressively throughout the training sessions. The mean increased steadily from the start to the end, suggesting an improvement in exercise capacity during the rehabilitation program. The 95% confidence intervals indicate moderate variability in the data, which tends to increase with the progression of the sessions, reflecting interindividual differences in response to the training. Starting with the intermediate sessions, a clearer separation from the initial values Thwas observed, suggesting a consistent increase in energy consumption. The most pronounced increase was observed towards the end of the program (moment VIII), where both the mean and the confidence interval were located at significantly higher values Ththan in the initial moment.

### The influence of gender on the energy consumed during MICT

Male gender was significantly associated with the energy consumption in both analyzed models. Thus, in the simple model, male gender was associated with a higher energy consumption than the female gender (β = 177; CI 95%, 95 to 260; *P* < 0.001), the result being also preserved in the multiple model (β = 172; CI 95%, 90 to 255; *P* < 0.001).

### The influence of LVEF on the energy consumed during MICT

The final multiple model indicates a positive association between the initial value of LVEF and the energy consumption, with each increase by 1% of the LVEF initial value being associated with an increase in energy consumption of 4.52 kJ (β = 4.52; CI 95%,1.55 to 7.48; *P* = 0.003), within the value interval noticed in the group (25–68%).

### The influence of max HR on energy consumption during MICT

In the multiple model, max HR showed a statistically significant positive association with energy consumption (β = 0.80; CI 95%, 0.42 to 1.18; *P* < 0.001), suggesting an increase in energy consumption with higher heart rate.

The energy efficiency (a derivative indicator) was used to estimate the efficiency of the response to effort, thus expressing the ratio between the energy consumed and the reached max HR:


$$energy\;efficiency=\frac{energy\;consumed\left[kJ\right]}{HR\;\left[bpm\right]}$$

Its increase may indicate a favorable adaptation to effort and improved cardiovascular efficiency.

We noticed an increase in energy efficiency, with the last session reaching a maximum value 1.23 times higher than the first (Table 8).

**Table 8:** The evolution of energy efficiency of patients depending on the aerobic training sessions on the sensor ergometric bicycle

	Energy efficiency [kJ/bpm]		
Predictors	Estimates	CI 95%	*P*
Moment [I]	1.88	1.55–2.20	<0.001
Moment [II]	0.12	-0.06–0.29	0.205
Moment [III]	0.31	0.13–0.48	0.001
Moment [IV]	0.42	0.24–0.60	<0.001
Moment [V]	0.65	0.47–0.83	<0.001
Moment [VI]	0.80	0.62–0.98	<0.001
Moment [VII]	0.80	0.63–0.98	<0.001
Moment [VIII]	1.23	1.05–1.41	<0.001
**Random Effects**			
σ^2^	0.17		
τ_00__ID_	0.96		
ICC	0.85		
N _ID_	43		
Observations	344		
Marginal R^2^ / Conditional R^2^	0.115 / 0.867		

### The influence of the other variables on energy consumption during MICT

Blood pressure, both systolic (SBP) and diastolic (DBP), was not significantly associated with the energy consumption in the simple model (SBP: β = -0.4; *P* = 0.28; DBP: β = -0.05; *P* = 0.91), nor in the multiple model (SBP: β = -0.3; *P* = 0.37; DBP: β = 0.3; *P* = 0.49). In both models, BMI did not have a statistically significant effect on energy consumption during training. Age was negatively and significantly associated with energy consumption in the simple model (β = -7.5; CI 95%: -12 to -3; *P* = 0.001), while this association became insignificant in the multiple model (β = -1.5; CI 95%: -6 to 3; *P* = 0.49).

### Results regarding the METs value registered during the training sessions

In the comparative analysis of the models for METs (Table 9), a progressive increase was highlighted throughout the training program. The differences from the initial moment became statistically significant starting with moment III, both in the simple model (β = 0.30; *P* = 0.016) and in the multiple model (β = 0.25; *P* = 0.041), maintaining until moment VIII (β = 1.1; *P* < 0.001).

**Table 9:** Simple models vs. complete multiple model regarding METs consumption

Predictor	Simple models			Multiple model			
	*n*	Beta (95% CI)	*P* value	Beta (95% CI)	*P* value	GVIF	Adj GVIF1
Moment	312					1.2	1.0
I		—		—			
II		0.00 (-0.25 to 0.25)	0.99	0.00 (-0.24 to 0.23)	0.98		
III		0.30 (0.06 to 0.55)	0.016	0.25 (0.01 to 0.48)	0.041		
IV		0.53 (0.28 to 0.78)	<0.001	0.48 (0.24 to 0.71)	<0.001		
V		0.66 (0.41 to 0.90)	<0.001	0.58 (0.34 to 0.81)	<0.001		
VI		0.81 (0.56 to 1.1)	<0.001	0.72 (0.48 to 0.96)	<0.001		
VII		0.94 (0.70 to 1.2)	<0.001	0.86 (0.62 to 1.1)	<0.001		
VIII		1.2 (0.99 to 1.5)	<0.001	1.1 (0.87 to 1.4)	<0.001		
Age (years)	312	-0.03 (-0.08 to 0.01)	0.14	0.01 (-0.04 to 0.06)	0.67	1.5	1.2
Gender	312					1.4	1.2
F		—		—			
M		1.3 (0.46 to 2.1)	0.003	1.3 (0.40 to 2.2)	0.006		
LVEF (%)	312	0.02 (-0.02 to 0.06)	0.26	0.03 (0.00 to 0.07)	0.060	1.1	1.1
Max HR (bpm)	312	0.02 (0.01 to 0.02)	<0.001	0.01 (0.01 to 0.01)	<0.001	1.1	1.1
SBP (mmHg)	312	0.01 (0.00 to 0.02)	0.046	0.01 (0.01 to 0.02)	0.001	1.4	1.2
DBP (mmHg)	312	0.01 (0.00 to 0.02)	0.17	0.00 (-0.01 to 0.01)	0.74	1.4	1.2
BMI (kg/m^2^)	312	-0.02 (-0.10 to 0.07)	0.73	-0.01 (-0.09 to 0.07)	0.76	1.1	1.1

In the final multiple model (Table 10), there was a progressive increase in METs that was statistically significant starting with Moment IV.

**Table 10:** The final multiple model for MET consumption, including significant predictors

	METs		
Predictors	Estimates	CI	*P*
Intercept (Moment I)	-1.32	-3.15 to 0.51	0.157
Moment [II]	-0.01	-0.24 to 0.22	0.935
Moment [III]	0.21	-0.01 to 0.44	0.065
Moment [IV]	0.42	0.19 to 0.65	<0.001
Moment [V]	0.52	0.29 to 0.74	<0.001
Moment [VI]	0.67	0.44 to 0.90	<0.001
Moment [VII]	0.81	0.58 to 1.04	<0.001
Moment [VIII]	0.98	0.74 to 1.21	<0.001
Gender [M]	1.16	0.46 to 1.86	0.001
LFEF	0.03	-0.00 to 0.06	0.064
Max HR	0.01	0.01 to 0.01	<0.001
SBP	0.01	0.01 to 0.02	<0.001
**Random Effects**			
σ^2^	0.24		
τ_00__ID_	0.71		
ICC	0.74		
N _ID_	39		
Observations	312		
Marginal R^2^ / Conditional R^2^	0.350 / 0.833		

The final multiple model for METs accounts for approximately 83% of the outcome variance, with approximately 35% accounted for by fixed effects (Marginal R^2^= 0.350) and approximately 48% by random effects (Conditional R^2^= 0.833). Interindividual variability was significant (ICC = 0.74).

The same progressive increase was also obvious when using descriptive data (Table 11), where the mean increased from 3.47 ± 1.12 at the starting moment (I) to 4.71 ± 1.39 at the final moment (VIII). A similar evolution was observed at the median level, which increased from 3.3 at time I to 4.6 at moment VIII. The minimum and maximum values Thindicate significant interindividual variability throughout the training program.

**Table 11:** The evolution of METs – descriptive data

	Moment	Average	SD	Median	Minimum	Maximum
1	I	3.47	1.12	3.3	1.8	6.8
2	II	3.47	1.12	3.3	1.8	6.8
3	III	3.77	1.31	3.4	1.8	8
4	IV	4	1.32	3.9	1.8	8
5	V	4.13	1.29	3.9	2.2	8
6	VI	4.28	1.24	4.2	2.2	7
7	VII	4.41	1.2	4.5	2.2	7.4
8	VIII	4.71	1.39	4.6	2.2	7.4

Male gender was significantly associated with higher METs in both the simple (β = 1.3; *P* = 0.003) and multiple (β = 1.3; *P* = 0.006) models. The max HR remained a significant predictor in both models (β = 0.02 vs. 0.01; *P* < 0.001).

SBP was marginally significant in the simple model (*P* = 0.046), but it became significant in the multiple model (β = 0.01; *P* = 0.001), indicating an independent effect after adjustment.

LVEF was not significant in the simple model (*P* = 0.26), but showed a tendency towards significance in the multiple model (β = 0.03; *P* = 0.060).

Age, DBP, and BMI were not significantly associated with METs values Thin any of the models (*P* > 0.05).

**Figure 5:**
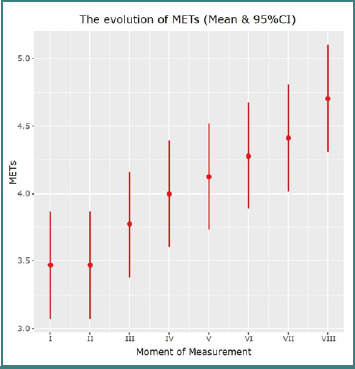
Changes in the METs value throughout the CVR program (mean and CI 95%)

In the graph shown in Figure 5, METs increased progressively throughout the training sessions. The mean increased steadily from the start to the end, suggesting an improvement in exercise capacity during the recovery program. The 95% confidence intervals indicate moderate variability in the data, which tends to increase as the sessions progress, reflecting interindividual differences in training response.

## DISCUSSION

Differences were observed between the values obtained through descriptive analysis and the estimates from the statistical models (simple and complete multiple) for the energy outcome. For example, in the descriptive analysis, the average difference between Moment I and Moment II was approximately 16 kJ, whereas the model-estimated coefficient for Moment II differs both between models and within the descriptive analysis (β ≈ 13–15 kJ, depending on the model used). These differences can be accounted for using statistical models that do not estimate simple raw differences between averages but rather adjusted effects. The models used account for interindividual variability and the repeated structure of the data, assigning different weights to observations based on their consistency.

Moreover, the coefficient values differ across the simple, multiple, and final models when different numbers of predictors are included. Thus, in the multiple model, the effect estimate was adjusted for the influence of other variables. In the final model, the additional differences reflect the selection of a subset of relevant predictors.

Therefore, variations in coefficient values between models are expected and reflect successive adjustments in estimates based on each model’s structure and complexity.

Compared with the simple models, the multiple model showed changes in the magnitude and direction of the coefficients for some predictors. For certain variables, the coefficient values decreased, and their effects became non-significant, whereas for others, such as LVEF, the coefficient values increased and became statistically significant. In some cases, the direction of association was reversed; for example, DBP showed a negative association in the simple model but a positive association in the multiple model. These changes suggest interdependence among the predictors and may indicate multicollinearity. This interdependence may explain the observed instability of coefficient estimates in the multiple model.

The baseline characteristics of the study sample are consistent with the frequent occurrence of coronary heart disease among young men. Data from the literature indicate that, between 1995 and 2014, approximately 30% of all acute myocardial infarctions occurred in individuals aged 35 to 54 years, the majority of whom were men [6].

The study group is representative of patients after myocardial infarction with an increased cardiovascular risk profile (smokers, overweight people), consistent with the data in the literature, which support the central role of prevention in the management of cardiovascular diseases [3]. These risk factors may be associated with the decreased tolerance to physical effort of patients after acute MI [7].

The concept of “energy” used in the study reflects energy consumption associated with physical effort. It serves as an indirect indicator of functional capacity and the efficiency of the cardiovascular response to exercise.

From a physiologic point of view, increased energy consumption during training suggests a better capacity to generate and sustain effort, which depends on the integration of several mechanisms: increased cardiac output, adaptation of heart rate, optimization of oxygen use at the muscular level, and the efficiency of energy metabolism [8].

Although energy consumption reflects mechanical work, it is indirectly related to metabolic demand and effort capacity, which are traditionally assessed using parameters such as max VO_2_ or METs. In this context, energy consumption can serve as a practical marker of effort capacity when direct metabolic measurements are unavailable.

### The increase in energy consumption according to the training sessions

The trend in energy consumption during MICT is relatively linear, without significant intermediate decreases, indicating good tolerance and adaptation to the training program.

The confidence intervals highlight moderate variability in the values, but the limited overlap between consecutive moments suggests real differences between the program stages. These observations are consistent with the results of the statistical models, which indicate a significant increase in energy consumption starting from the intermediate moments of the program.

The high ICC indicates that a significant proportion of the total variability is accounted for by interindividual differences, suggesting a hierarchical structure of the data and relevant clinical heterogeneity among patients.

The progressive increase in the energy consumed by the patients during the training program by pedaling on the sensor ergometric bicycle, which occurred with session 3 and was observed until the end of the program, suggests that interventions based on structured physical exercise contribute significantly to the improvement of cardiovascular functional performance and energy in intermediary metabolism, an aspect consistent with the data in the specialty literature on the efficiency of CVR programs [9].

After reviewing the relevant literature, we identified the third training session as a key point at which physiological adaptation to physical effort may begin. A recent study conducted in patients with a first acute MI who underwent CVR reported that, during the third training session, “the most important metabolic adaptation occurs mainly at the level of energy metabolism pathways,” suggesting increased oxidative energy production and improved exercise performance [10]. Physical exercise may also have favorable effects on mitochondrial function [11].

Moderate-intensity continuous training was associated with a significant increase in the activity of antioxidant enzymes, including superoxide dismutase 1 (SOD1) and superoxide dismutase 2 (SOD2), as well as with an improvement in the reduced glutathione to oxidized glutathione ratio (GSH/GSSG), an important marker of cellular redox balance. These effects have been attributed to the activation of mitochondrial protective mechanisms, particularly through exercise-induced deacetylation of sirtuin 3 (SIRT3) [12].

Through these mechanisms, physical training reduces oxidative stress, optimizes mitochondrial function, and improves the functional capacity of patients enrolled in CVR programs [11].

Patients showed an average increase in energy of 184.10 kJ and also an increase in METs of 1.24. The initial values Thof energy consumption (approximately 223 kJ) were in the lower range of intervals associated with light physical activities, close to the metabolic rate at rest (approximately 1 MET), suggesting a very low level of functional demand at the beginning of the program and reached an average level of energy consumption of approximately 407.91 Kj, corresponding to a light intensity physical effort according to Table 12, placed in the lower range of aerobic activities, such as those falling within the 3-4 METs range (e.g. 30 minutes of Pilates training, volleyball). The favorable evolution of the energy quantified during the hospitalization period suggests that this tolerance to effort will likely increase at home, possibly modulated by telemedicine [13].

**Table 12:** Examples of physical activities, corresponding metabolic equivalent values, and estimated energy consumption

Activity type	MET value	Energy consumption (cal)	Energy consumption (kJ)
At rest (sitting or lying)	1 MET	60 cal	251 kJ
**Exercises (30 minutes)**			
Pilates	3.5 METs	105-131 cal	439-548
Fast walking 4.8 km/h	5 METs	150-188 cal	628-787
Weight lifting	6 METs	180-225 cal	753-942
Crawl swimming	8 METs	240-300 cal	1004-1256
Calisthenics (rhythmic gymnastics)	8 METs	240-300 cal	1004-1256
Running 9 minutes	11 METs	330-413 cal	1381-1729
**Household activities (30 minutes)**			
Carpet vacuum	3.3 METs	99-124 cal	414-519
Gardening	4 METs	120-150 cal	502-628
Activities with the pet (dog)	4 METs	120-150 cal	502-628
Washing and polishing the car	4.5 METs	135-168 cal	565-703
Playing with children	5 METs	150-188 cal	628-787
Furniture moving	6 METs	180-225 cal	753-942
**Sports activities**			
Volleyball	3 METs	90-113 cal	376-473
Golf	4.5 METs	135-169 cal	565-707
Dancing	4.8 METs	144-180 cal	602-753
Backpacking	7 METs	210-263 cal	879-1100
Basketball	8 METs	240-300 cal	1004-1255
Football	10 METs	300-375 cal	1255-1569

Adapted from [13] with modifications.

The low initial level of physical exercise intensity denotes that training was initiated at a submaximal level under precautionary conditions in patients after acute MI, in accordance with current guidelines.

The graphical analysis of the evolution of functional parameters (Figure 4 and Figure 5) highlights differences in the dynamics of MET values Thand energy consumption. Thus, although both variables showed an upward trend during the training program, the increase in energy consumption was more pronounced than that in MET values.

### The influence of the male gender on energy consumption

The difference in energy consumption between genders may be influenced by factors such as body composition, training status, and mechanisms of adaptation to physical effort. The reasons for higher energy consumption during training in men than in women are not yet fully elucidated. However, these differences in energy metabolism may be explained by several factors, including higher body mass in men, which implies a greater amount of metabolically active tissue and, consequently, higher energy consumption; higher cardiac output, stroke volume, and maximal oxygen consumption (VO_2_max), which may allow men to sustain greater energy expenditure at the same LVEF value; and hormonal differences, including the effects of sex steroids, insulin resistance, and leptin metabolism, which contribute to higher body fat levels in women compared with men and, consequently, a lower capacity for energy consumption due to a reduced lean mass to fat mass ratio [14].

### The increase in energy consumption according to LVEF

The results obtained are in accordance with the data in the specialty literature, which support the fact that a higher LVEF value may reflect more efficient myocardial contractility and a cardiac output adequate to the demand of the muscles undergoing contractile activity, facilitating the delivery of oxygen and energy substrate to them and the consequent increase in energy consumption within MICT [15,16].

### The increase in energy consumption according to max HR

The positive relationship identified between max HR and energy consumption may reflect greater involvement of adaptive physiological mechanisms during effort [17].

The increase in energy output during training sessions may suggest improved cardiovascular efficiency. Thus, at the beginning, the deconditioned patient after acute MI can sustain the effort through an accentuated chronotropic response (tachycardia). At the same time, later, for similar heart rate values, they manage to sustain training at higher intensity/duration, resulting in greater energy expenditure. This evolution reflects a favorable adaptation to the effort, with increased efficiency of the cardiovascular system and peripheral oxygen utilization.

In contrast, SBP, DBP, and average BP did not show statistically significant associations with energy consumption. This result can be explained by the fact that blood pressure rather reflects the hemodynamic status at the moment, without directly influencing energy consumption in the analyzed conditions. This finding highlights a key difference in adaptive response between individuals without a history of acute MI and patients after acute MI, as SBP increases proportionally with intensity and energy consumption in the former group, reflecting increased cardiac output [18].

### Limitations

Although max HR can be considered a significant predictor, the likely presence of additional unmeasured factors should be acknowledged as a limitation of the model used. These factors may include metabolic variables, such as blood glucose levels and lipid profile; individual or behavioral variables, such as baseline pre-rehabilitation training level, compliance with the rehabilitation program, and patient motivation; and measurement-related variables, such as time of day, chronorhythmicity, infarction severity, and revascularization status.

Most participants performed their training sessions at the same time of day, which helped reduce circadian variability in the monitored parameters. However, this was not a uniform feature across all patients.

These factors may influence energy consumption, making it appropriate to consider them in future studies, including from the perspective of more tailored and personalized approaches within CVR programs.

The number of observations available for the analysis of MET values was slightly lower than that for energy consumption (5 patients fewer), due to the unavailability of these data recorded by the equipment in certain sessions.

The relatively small sample size may affect the statistical power and reliability of the estimates. Although 352 observations were obtained through repeated measurements at eight distinct time points, these were not independent because they came from the same participants, limiting the increase in statistical power. Moreover, the relatively small sample size did not allow for finer age-group stratification. The division into more detailed categories led to uneven distributions, with some subgroups comprising only a few or even zero patients.

This analysis includes a limited set of initial sample characteristics relevant to the current objectives.

Some of the patients included in the study had an acute MI in other medical centers and were subsequently referred to our department for CVR. This lack of complete access to the database from the originating centers led to missing values for some variables (e.g., revascularization status).

Given the significant heterogeneity among patients, the results must be interpreted with caution as reflections of the associations and rehabilitation dynamics, without allowing the establishment of causal relationships.

The practical implications of these results suggest the usefulness of periodic monitoring of parameters measured by the sensor-ergometric bicycle to dynamically adjust the intensity and structure of CVR programs. The significant interindividual variability observed supports the need for an individualized approach, adapted to the initial clinical characteristics and the response to the intervention. In this sense, standardized protocols should allow for the personalization of interventions as each patient’s condition evolves. Also, early identification of patients with suboptimal response could help optimize therapeutic strategies. However, the clinical applicability of these observations requires confirmation in future controlled trials with larger samples.

### Future prospectives

Future randomized studies on larger samples, with data collected from more than two clinics, are needed to overcome an inherent limitation of our current research, if the concept of CVR is to gain the necessary amplitude, as there are currently few CVR centers nationwide. A standardized CVR protocol and a national electronic registry for CVR are needed to facilitate access to data that could be the basis for future studies. With the development and modernization of hospital infrastructure, it is expected that comprehensive assessment and individualization of exercise capacity can be performed, surpassing that offered by cardiopulmonary exercise testing (CPET), allowing for a more precise characterization and optimization of recovery programs.

## Conclusion

The initiation and implementation of the CVR program were interventional, interdisciplinary, and multiprofessional. This study revealed a progressive increase in energy consumption and METs within the CVR program, with significant changes occurring after the initial adaptation phase. The observed interindividual variability underscores the importance of a patient-adapted approach, including modulation and short-, medium-, and long-term monitoring. With these results, our study not only supports the role of interdisciplinary collaboration in general but also specifically highlights the need for individualized monitoring and progressive adjustment of effort intensity within CVR programs.

This statistical analysis is valuable for providing a detailed characterization of the dynamics of the response to the rehabilitation intervention, providing partial data relevant to protocol optimization and substantiating subsequent comparative analyses.
